# Oral and intratumoral microbiota influence tumor immunity and patient survival

**DOI:** 10.3389/fimmu.2025.1572152

**Published:** 2025-05-21

**Authors:** Kaitong Wei, Yaqing Ma, Jing Xu, Shuijuan Hu, Hongyu Zheng, Yuelian Liu, Qiang Sun

**Affiliations:** ^1^ Oral and Maxillofacial Surgery, The First Affiliated Hospital of Zhengzhou University, Zhengzhou, Henan, China; ^2^ Department of Oral Cell Biology, Academic Center for Dentistry Amsterdam (ACTA), Vrije Universiteit Amsterdam and University of Amsterdam, Amsterdam, Netherlands

**Keywords:** OSCC, 16s rDNA sequencing, transcriptome, oral microbiota, Capnocytophaga, immune responses

## Abstract

**Background:**

The aim of this study was to analyze the changes in the oral microbiota of patients with oral squamous cell carcinoma (OSCC) compared to healthy controls and the effect of intratumoral microorganisms on the host immune microenvironment.

**Methods:**

Saliva samples were collected from 36 OSCC patients and 34 healthy controls. 16S rDNA sequencing and bioinformatics analysis were conducted on the saliva samples. Differential expression, pathway enrichment, and tumor microenvironment analyses were performed on transcriptome data from head and neck squamous cell carcinoma (HNSCC) in The Cancer Genome Atlas (TCGA) database and OSCC patients in the GEO database.

**Results:**

Oral microbiota exhibited comparable α diversity but distinct β diversity between OSCC patients and healthy controls. *Capnocytophaga*, *Flavobacteriaceae*, and *Vibrionaceae* were significantly enriched in the OSCC group. Pathway analysis revealed dysregulation of metabolic pathways, including arginine and proline metabolism and sulfur transfer systems, in the OSCC group. The presence of microorganisms activated immune responses within tumor tissues, and immune scores increased with disease progression. Changes in the abundance of tumor immune-related signaling pathways were significantly associated with patient survival.

**Conclusion:**

Specific oral microbiota in OSCC patients may serve as biomarkers for distinguishing OSCC. The interaction between microorganisms and the host alters the tumor immune microenvironment, which provides a theoretical basis for OSCC immunotherapy.

## 1 Introduction

OSCC is a malignant tumor that occurs in the squamous epithelium of the complex layers of the oral mucosa, and is strongly associated with exposure to risk factors such as alcohol consumption, smoking, betel nut chewing (1–3). According to recent epidemiologic estimates, there are approximately 389,000 new cases of OSCC and 188,000 deaths globally each year (4). The insidious onset of OSCC leads to a majority of patients being diagnosed at advanced stages (III or IV). Despite continuous advances in diagnostic and therapeutic strategies, the 5-year survival rate of OSCC is still only about 50%, which is mainly due to the late diagnosis, frequent relapses, and lack of clarity about the etiology of the disease (5, 6). Given the aggressive progression of OSCC, the heavy global burden, and the current lack of clarity on its etiology, there is an urgent need to elucidate the mechanisms of OSCC occurrence and development.

Microbial communities in various anatomical niches exhibit resilience against perturbations, enabling them to return to baseline states over time. There are altered community structures in various disease states called dysbiosis, where harmful microbiota increase. These processes drive metabolic dysregulation, generating pathogenic metabolites and antigens that trigger maladaptive immune-inflammatory cascades-key mechanistic pathways implicated in carcinogenesis (7, 8). Epidemiological studies attribute approximately 20% of malignancies to microbial etiology, with pathogens driving oncogenesis through genomic instability induction via compromised DNA repair, cell cycle dysregulation, proliferative signaling activation, and apoptotic pathway suppression (9, 10).

Studies have reported a strong association between *Clostridium difficile* and colorectal cancer, periodontal disease, halitosis, oral cancer, breast cancer and rheumatoid arthritis (11–15). *Escherichia coli* exerts tumorigenic effects in the colonic mucosa affecting colorectal carcinogenesis and progression (16). *Porphyromonas gingivalis* as an oral pathogen strongly associated with pancreatic cancer and OSCC (17, 18). Al-Hebshi et al. showed that *Pseudomonas aeruginosa* and *F. nucleatum* were associated with OSCC development (19). In addition, significant changes in the oral microbiota have been reported as OSCC progresses and the relative abundance of specific bacterial species increases (20, 21). While second only to the gut microbiome in microbial diversity, oral dysbiosis demonstrates systemic pathophysiological implications, being mechanistically linked to multiple malignancies (esophageal, pancreatic, colorectal, gastric, lung), neurodegenerative disorders including Alzheimer’s disease, and cardiovascular pathologies (22–28). Despite advances in understanding microbial associations with OSCC, the mechanistic impact of oral microbiota on tumor immunity remains underexplored (29).

In this study, we collected saliva samples from oral squamous cell carcinoma and healthy control subjects, and analyzed the correlation between saliva microbial diversity and clinical pathological characteristics of OSCC patients using 16S rDNA sequencing. In addition, we further analyzed the changes in host transcription and immune signaling pathways influenced by microorganisms in OSCC tissues using data from published studies by others, provide a theoretical framework for understanding the interaction between OSCC and the oral microbiota.

## 2 Materials and methods

### 2.1 Study participants

36 cases of OSCC and 34 healthy volunteers were recruited at the Department of Stomatology of the First Affiliated Hospital of Zhengzhou University from October 2022 to June 2023. All participants provided informed consent and had complete clinical and pathologic information. The study was approved by the Ethics Committee of the First Affiliated Hospital of Zhengzhou University (the Ethics Approval: 2019-KY-305).

Inclusion criteria for the OSCC group: (1) Patients with pathological biopsy of OSCC; (2) Patients without other serious oral diseases such as severe periodontal disease, severe caries, and other systemic diseases in the 3 months prior to enrollment; (3) No history of surgery, no history of antibiotic application, and no history of radiotherapy. OSCC exclusion criteria: (1) History of oral infectious diseases or bleeding; (2) History of antibiotic use within 3 months before enrollment; (3) History of other systemic diseases were excluded. The OSCC cases included in this study were strictly limited to primary foci in the oral cavity, excluding cases involving the oropharynx. Healthy participants: (1) No antibiotic use within 3 months, no history of oral infectious disease or bleeding; (2) No history of severe periodontal disease, severe caries or other serious oral diseases; (3) No personal or family history of autoimmune diseases or other serious systemic diseases were included in the Healthy control (HC) group.

### 2.2 Sample collection

Saliva samples were collected between 8:00 and 9:00 AM. Each participant was asked to refrain from smoking, drinking, or eating for at least 1 hour before sample collection. The mouth was rinsed twice with distilled water and participants were instructed to gently press the tip of the tongue against the lingual side of the palate or mandibular incisors to enrich the saliva, which was then gently spat into a centrifuge tube until the liquid saliva (non-bubbly) reached the 5 ml mark. Centrifugation was performed at 4°C and 1,006 x g for 10 min and the supernatant was dispensed into new EP tubes. Immediately after coding, store in an ultra-low temperature refrigerator (-80°C) for backup.

### 2.3 DNA extraction and 16S rDNA sequencing of saliva samples

Microbial genomic DNA was extracted from saliva samples using the cetyl trimethyl ammonium bromide (CTAB) method. The lysate was enriched with lysozyme and Proteinase K to ensure that the cell walls of Gram-positive bacteria could be lysed during sample processing. The DNA purity was assessed using a NanoDrop One spectrophotometer, and its integrity was verified via 1% agarose gel electrophoresis. The DNA was then diluted to 1 ng/μl, and the 16S rDNA of the V3-V4 region was amplified using primers 341F (5’-CCTACGGGNGGCWGCAG-3’) and 805R (5’-GACTACHVGGGTATCTAATCC-3’) (30). PCR products were purified using AMPure XP magnetic beads (Beckman Coulter Genomics, Danfoss, MA, USA) and quantified by Qubit (Invitrogen, Carlsbad, CA, USA). PCR amplified products were detected by electrophoresis on a 2% agarose gel and recovered using the AMPure XP magnetic Bead Recovery Kit (Beckman, USA). Purified PCR products were evaluated using an Agilent 2100 Bioanalyzer (Agilent, USA) and an Illumina Kapa Biosciences (Wolborn, MA, USA) library Quantification kit, and qualified library concentrations were above 2nM. For each qualified library (Index sequence was not repeatable), the library was diluted in gradient according to the required amount of sequencing, mixed in appropriate proportion according to the required amount of sequencing, and then denatured to single-strand sequencing by sodium hydroxide. 2×250 bp double-ended sequencing was performed on a NovaSeq6000 sequencer (Illumina, USA) using the corresponding reagent NovaSeq6000SP kit (500 cycles). Cutadapt (v1.9) software was used to remove primer sequences and balanced base sequences of Raw Data. After that, each pair of paired-end reads was spliced into a longer tag using FLASH (v1.2.8). The default scanning window was 100bp. When the average quality value in the window was lower than 20, the part of the read from the start to the 3’ end of the window was truncated. Finally, the truncated sequences less than 100bp in length, the truncated sequences with more than 5% N (uncertain fuzzy base) and the chimeric sequences (software: Vsearch (v2.3.4)) were removed. After DADA2 denoising and clustering, the required Amplicon Sequence Variants (ASVs) representative sequences and abundance tables were obtained for subsequent analysis (software and module: Quantitative Insights into Microbial Ecology (QIIME) dada2 denoise-paired).

### 2.4 Microbiota analysis, statistical analysis and visualization

In 16S rDNA gene sequencing analysis, α and β diversity values were calculated using QIIME toolkit (version 2.0). After refining the Operational Taxonomic Unit (OTU) table, α-diversity was used to calculate species richness and evenness within bacterial populations using metrics such as Chao1, Observed species, Goods coverage, shannon, Simpson, and pielou-e indices. Wilcoxon paired signed rank test was used to calculate the significance of α diversity metrics. The heterogeneity of microbial communities, i.e. β-diversity, was determined using the unweighted UniFrac calculated by the QIIME script, and its significance was also determined by the aligned multivariate analysis of variance (ADONIS). The larger the UniFrac distance, the less similar the microbial communities were. Principal Coordinate Analysis (PCoA) was used to visualize differences in microbial distribution between individuals and/or groups. The relative abundance of oral microbial phyla and genera of the two groups of samples was statistically analyzed using linear discriminant analysis of effects (LEfSe). Only colonies satisfying both Linear Discriminant Analysis (LDA) values >3.5 and False Discovery Rate (FDR) values <0.05 were considered significantly enriched. The group performed Spearman correlation analysis using the relative abundance of differential microbiome at the phylum and genus levels with 10 clinical characteristics of OSCC patients: smoking, alcohol consumption, betel nut chewing, Body Mass Index (BMI), degree of tumor differentiation, TNM stage, clinical stage, lymph node metastasis, and neural invasion. At the phylum level, the FDR <0.05 and |log_2_FC| >0.5. At the genus level, the differential microbiome was restricted to satisfy the FDR <0.05 and |log_2_FC| >2. Stratified analyses were performed using the Union for International Cancer Control (UICC) 8th edition of Tumor-lymph node-metastasis (TNM) classification with Clinical stage, to investigate the relationship between tumor stage and the relative abundance of differential bacterial communities. In the present study, none of the patients had metastases. To predict the phenotypic and functional pathways of bacterial communities, we used Phylogenetic Investigation of Communities by Reconstruction of Unobserved States 2(PICRUSt2, https://github.com/respectively picrust/picrust2/, accessed August 30, 2024).

### 2.5 Transcriptome differential expression and enrichment analysis

Transcriptome data were sourced from TCGA’s head and neck squamous cell carcinoma cohort and GEO datasets GSE227919. Data cleaning and sample stratification were performed, followed by differential expression analysis using DESeq2 with raw counts. Significance was set at a fold change > 2 and Qvalue < 0.05. Enrichment analysis was conducted using clusterProfiler (v4.14.4) with hypergeometric tests and visualized using GseaVis (v0.0.5) and GSEABase (v1.68.0) bar charts.

### 2.6 Immune infiltration and immune scoring analysis

The CIBERSORT R script (version 1.03) was utilized for the analysis of immune infiltration within the TCGA dataset. For the GSE227919 dataset, immune infiltration analysis was conducted using the TIMER online database. Additionally, the ESTIMATE algorithm was employed to predict the proportion of infiltrating stromal and immune cells in tumor tissues.

### 2.7 Survival analysis

Survival data of OSCC patients were obtained from the Xena portal. Data cleansing and integration were performed prior to conducting survival analysis on oncological patients using the survival (version 3.8-3) and survminer (version 0.5.0) packages in R software (version 4.4.2).

### 2.8 Statistical analysis and data visualization

All figures and tables were generated using R software (version 4.4.2). Statistical significance of differences in boxplot analyses was assessed using either rank sum tests or t-tests. The notation “ns” indicates a p-value>0.05, “*”, “**”, and “***” represent 0.01<P<0.05, 0.01<P<0.001 and P <0.001, respectively.

All statistical tests were conducted as one-tailed test. Visualization was performed using the ggplot2 package.

## 3 Results

### 3.1 Clinical information statistical analyses of OSCC patients

From November 2022 to April 2023, the group recruited 40 patients diagnosed with OSCC as the OSCC group and 40 healthy controls as the HC group. The samples were subjected to in-depth bioinformatics analysis during the preliminary analysis stage of the sequencing data to identify and remove possible contaminating sequences or chimeras to ensure the accuracy of the final analysis results. Through these measures, 36 OSCC patients and 34 healthy controls were finally included in the study, and there was no marked difference in gender, age, weight, height, and BMI (Body Mass Index) between the two groups (P>0.05) (**Table 1**).

**Table 1:** Demographic and clinical characteristics for OSCC and HC groups.

	OSCC Group (n=36)	HC Group (n=34)	P
Sex (male/female)	18/18	16/18	0.710
Age	58.3 ± 14.6	56.9 ± 15.4	0.569
Weight/kg	64.5 ± 13.0	62.0 ± 12.6	0.290
Height/cm	168.2 ± 8.7	169.1 ± 8.9	0.668
BMI/(kg/m²)	23.9 ± 3.7	22.6 ± 2.6	0.154

A P-value < 0.05 was considered statistically significant.

Additionally, we examined the differences in clinical indicators such as smoking, alcohol consumption, betel nut chewing, tumor staging, and lymph node metastasis among 36 OSCC patients, with results presented in the **Supplementary File** (**Supplementary Table 1**). Our findings revealed that the mean age of OSCC patients who chewed betel nut was 38.8 years, whereas the mean age of those who did not was 60.1 years (**Table 2**).

**Table 2:** Betel nut chewing.

Name	Levels	0 (N=32)	1 (N=4)	P
Age	Mean ± SD	60.1 ± 14.7	38.8 ± 7.1	0.008
Gender	female	18 (56.2%)	0 (0%)	0.112
	male	14 (43.8%)	4 (100%)	
BMI	Mean ± SD	23.5 ± 3.8	25.5 ± 4.7	0.335
Smoking	0	23 (71.9%)	0 (0%)	0.023
	1	9 (28.1%)	4 (100%)	
Drinking	0	26 (81.2%)	2 (50%)	0.436
	1	6 (18.8%)	2 (50%)	
Differentiation.degree	0	3 (9.4%)	0 (0%)	0.522
	1	6 (18.8%)	0 (0%)	
	2	20 (62.5%)	4 (100%)	
	3	3 (9.4%)	0 (0%)	
Stage	1	7 (21.9%)	1 (25%)	0.880
	2	12 (37.5%)	2 (50%)	
	3	4 (12.5%)	0 (0%)	
	4	9 (28.1%)	1 (25%)	
Lymph.node.metastasis	0	22 (68.8%)	3 (75%)	1.000
	1	10 (31.2%)	1 (25%)	
Nerve.invasion	0	24 (75%)	2 (50%)	0.645
	1	8 (25%)	2 (50%)	

A P-value < 0.05 was considered statistically significant.

### 3.2 Differences in the composition of characteristic microbiota associated with oral cancer

**Figure 1:**
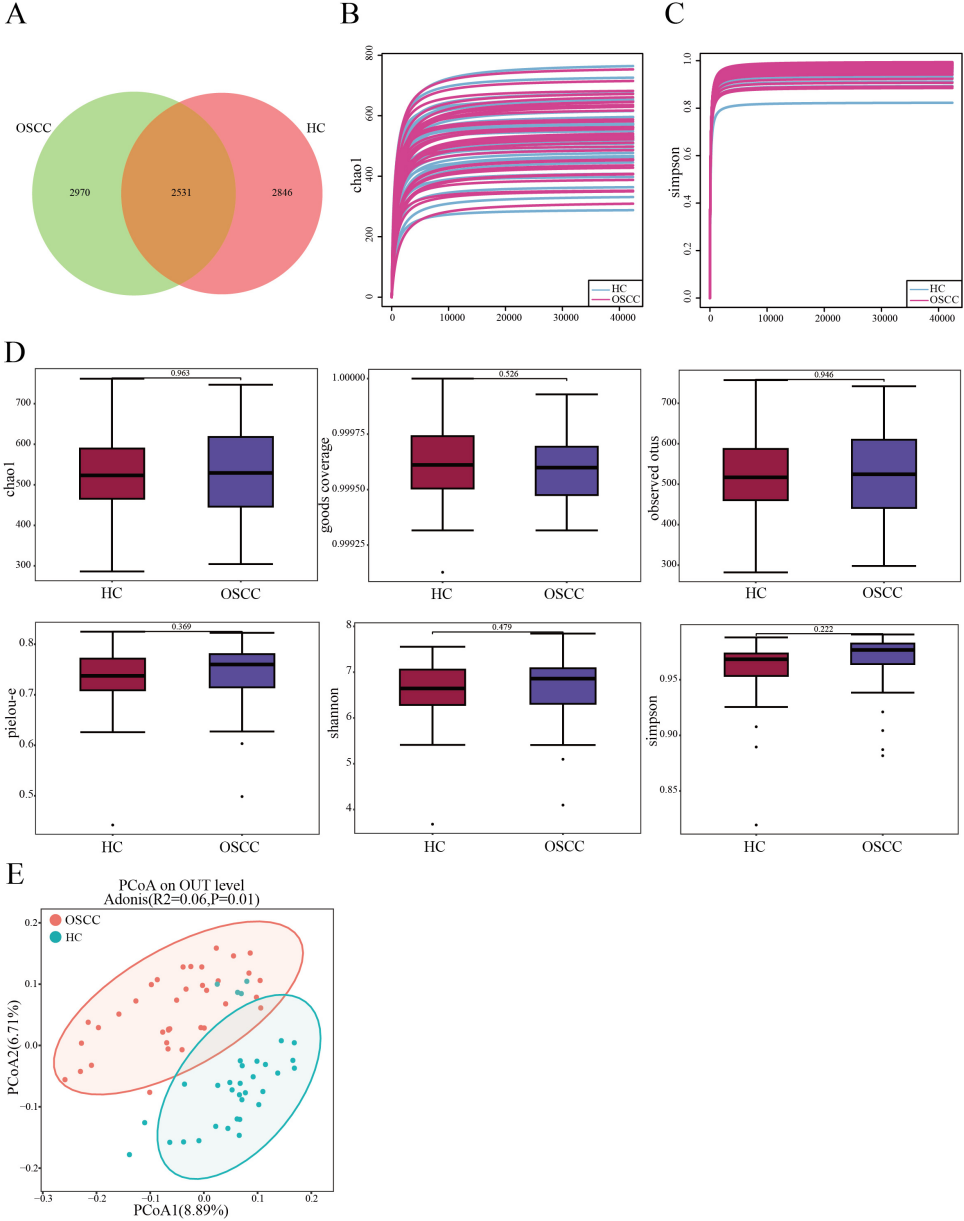
Comparisons of oral microbial diversity between OSCC and HC groups. **(A)** Venn diagram showing the distribution of ASVs in the two groups; **(B, C)** Sequencing depth of samples in OSCC and HC groups assessed using chao1 and Simpson’s Index; **(D)** Chao1, Goods coverage, Observed species, Pielou-e, Shannon and Simpson indices measure α diversity in OSCC and control groups; **(E)** β diversity between the two groups was calculated by PCoA based on unweighted unifrac distance.

In order to study the diversity of oral microbiome in OSCC patients, the group analyzed the microbial communities in the saliva of samples from both groups using 16S rDNA gene sequencing. ASVs shared between groups accounted for >45% of total abundance between the two groups. A total of 2,531 ASVs between the two groups, with 2,846 unique ASVs in the control group, and 2,970 unique ASVs (**Figure 1A**). The dilution curves obtained from the chao1 index and simpson’s index tended to be flat, indicating that the sequencing data were reasonable and of sufficient depth to represent the majority of the microbial species sequenced in this 16S rDNA sequencing (**Figures 1B, C**). P-values were calculated using the Wilcoxon rank-sum test, and the results of the analysis of each index of α diversity showed that the differences in α diversity between the two groups were not statistically marked (P>0.05) for chao1, Goods coverage, Observed species, Pielou-e, Shannon and Simpson indices (**Figure 1D**). There was no statistically significant difference in the α diversity of the colonies between the two groups (P>0.05), suggesting that there may not be marked differences in overall microbiome richness and evenness between the two groups. β diversity analyses were conducted to find out whether there were significant differences between the two groups. β diversity was first assessed based on Principal Coordinates Analysis (PCoA) of unweighted unifrac distances to explore the similarities and differences in community composition between the different groups. The results of the PCoA analysis showed that the samples between the OSCC group and the HC group were farther away from each other, and the differences in the species composition were larger, indicating that the significant changes in the microbiome between the two groups (**Figure 1E**). Subsequent non-parametric multivariate analysis of variance adonis test results under this distance matrix similarly showed statistically marked differences between the microbiota of the OSCC group and the control group (R^2^ = 0.06, P = 0.001). This suggests differences in the relative abundance of certain bacterial community between the two groups.

### 3.3 Abundance analysis of characteristic oral cancer-associated microbiome

**Figure 2:**
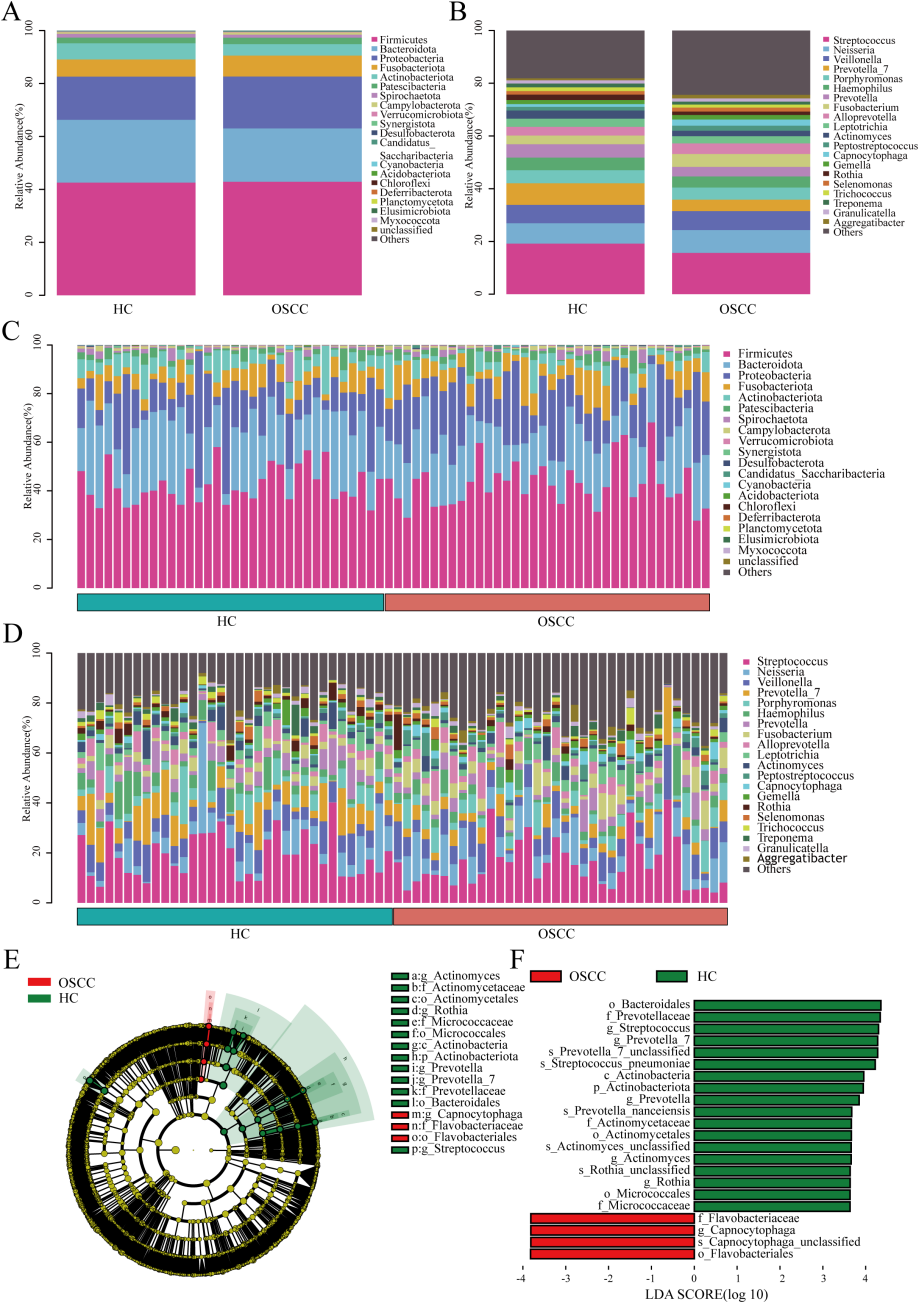
Composition and abundance of oral microbiome from OSCC and HC groups. **(A)** Column stacking plots of OSCC and HC groups at the phylum level, showing only the first 20 phyla; **(B)** Column stacked plot of OSCC and HC groups at the genus level, showing only the first 20 genera; **(C)** Bar-stacked plot of each sample at the phylum level for the OSCC and HC groups, showing only the first 20 phyla; **(D)** Stacked plot of bars at genus level for each sample in the OSCC and HC groups, showing only the first 20 genera; **(E)** The different circular layers radiating from inside to outside in the evolutionary branching diagram represent the different taxonomic levels of kingdom, phylum, class, order, family, genus, and species, and each node represents the classification of a taxa at that level, and the higher the abundance, the larger the node. Yellow nodes indicate that there is no significant difference between the two groups; red nodes indicate that there is a significant difference between the two groups and the species is enriched in the OSCC group; green nodes indicate that there is a significant difference between the two groups and the species is enriched in the HC group; **(F)** Linear discriminant analysis (LDA>3.5, P<0.05) showed that the OSCC group was differently enriched in the oral microbiome compared to the HC group.

Observed at different levels (phylum, class, order, family, genus, and species), the two groups had similar composition of dominant microbiota at each level, but different relative abundance. At the phylum level, the dominant phyla between the two groups were *Firmicutes* and *Bacteroidota*, but there was no significant difference (P>0.05). At the genus level, the dominant genera between the two groups were *Streptococcus* and *Neisseria*, and *Streptococcus* was significantly lower in the saliva of OSCC patients (P<0.05). While *Neisseria* had no marked difference between the two groups (P>0.05) and the dominant bacterial microbiome of the two groups are shown in **Figures 2A-D**. To elucidate the relative abundance of the dominant bacteria among the OSCC groups, this study adopted the LEfSe method with the screening criteria of LDA >3.5. The screened differential microbiome of different categories (phylum, class, order, family, genus, and species) in the OSCC group and HC group were enriched as shown in **Supplementary Table 2**. LEfSe analysis suggested that at the phylum level, the *Actinobacteriota* phylum was lower in the saliva of the OSCC group than that of the HC group (P<0.05). At the genus level, the enrichment level of genera such as *Capnocytophaga* was significantly higher in the saliva of the OSCC group compared with that of the HC group. Whereas genera such as *Streptococcus* and *Prevotella_7* were significantly less than that of the HC group (P<0.05) (**Figures 2E, F**). Taken together, these findings suggest a biased fitness of the oral microbiome of OSCC.

### 3.4 Correlation analysis between oral microbiome and clinical characteristics of OSCC patients

**Figure 3:**
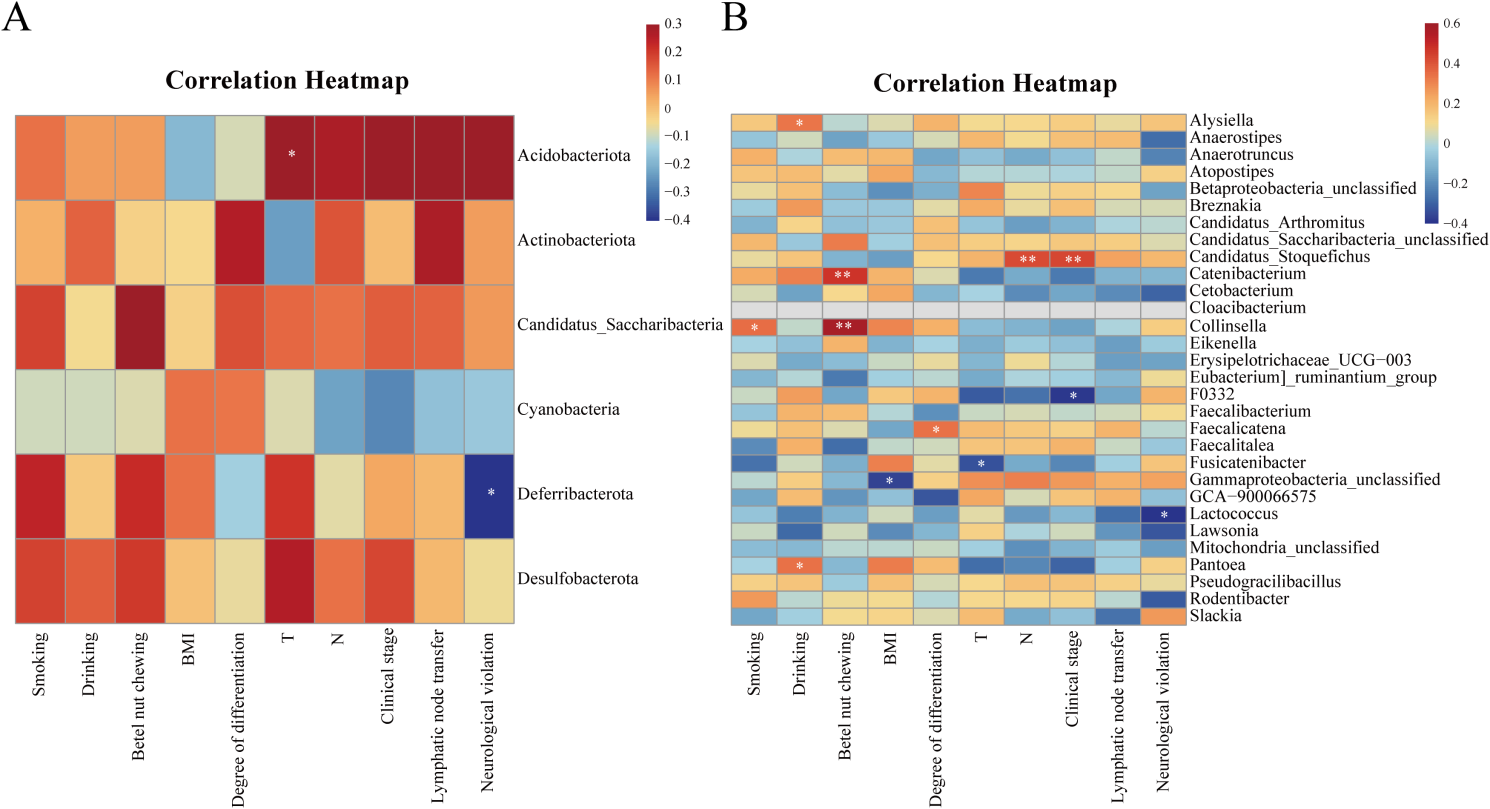
Heatmap of the correlation between the differential microbiome between the two groups and the clinical characteristics of the OSCC group. **(A)** Heatmap of correlation between differential microbiome and clinical characteristics between the two groups at the phylum level; **(B)** Heatmap of the correlation between differential microbiome and clinical characteristics between the two groups at the genus level. Red represents positive correlation and blue represents negative correlation, the stronger the correlation, the darker the colour. *P<0.05, **P<0.01.

To explore the relationship between oral microbiome and clinical characteristics of the OSCC group, this study correlated clinical characteristics such as OSCC lymph node metastasis and neurological violation with the top-ranked differential microbiome of the two groups at both the phylum and genus levels. At the phylum level, *Deferribacterota* was negatively correlated with neurological violation (P = 0.01, R = -0.41). *Acidobacteriota* was positively correlated with T stage (P = 0.03, R = 0.34) (**Figure 3A**). At the genus level, *Collinsella* and *Catenibacterium* were positively correlated with betel nut chewing (P<0.01, 0.4<R<0.6). *Candidatus stoquefichus* was positively correlated with clinical stage and N stage (P<0.01, 0.4<R<0.6). While *Lactococcus* had a negative correlation with neurological violation (P = 0.01, R = -0.41) (**Figure 3B**).

### 3.5 Prediction of functional pathways associated with OSCC characteristic microbiome

**Figure 4:**
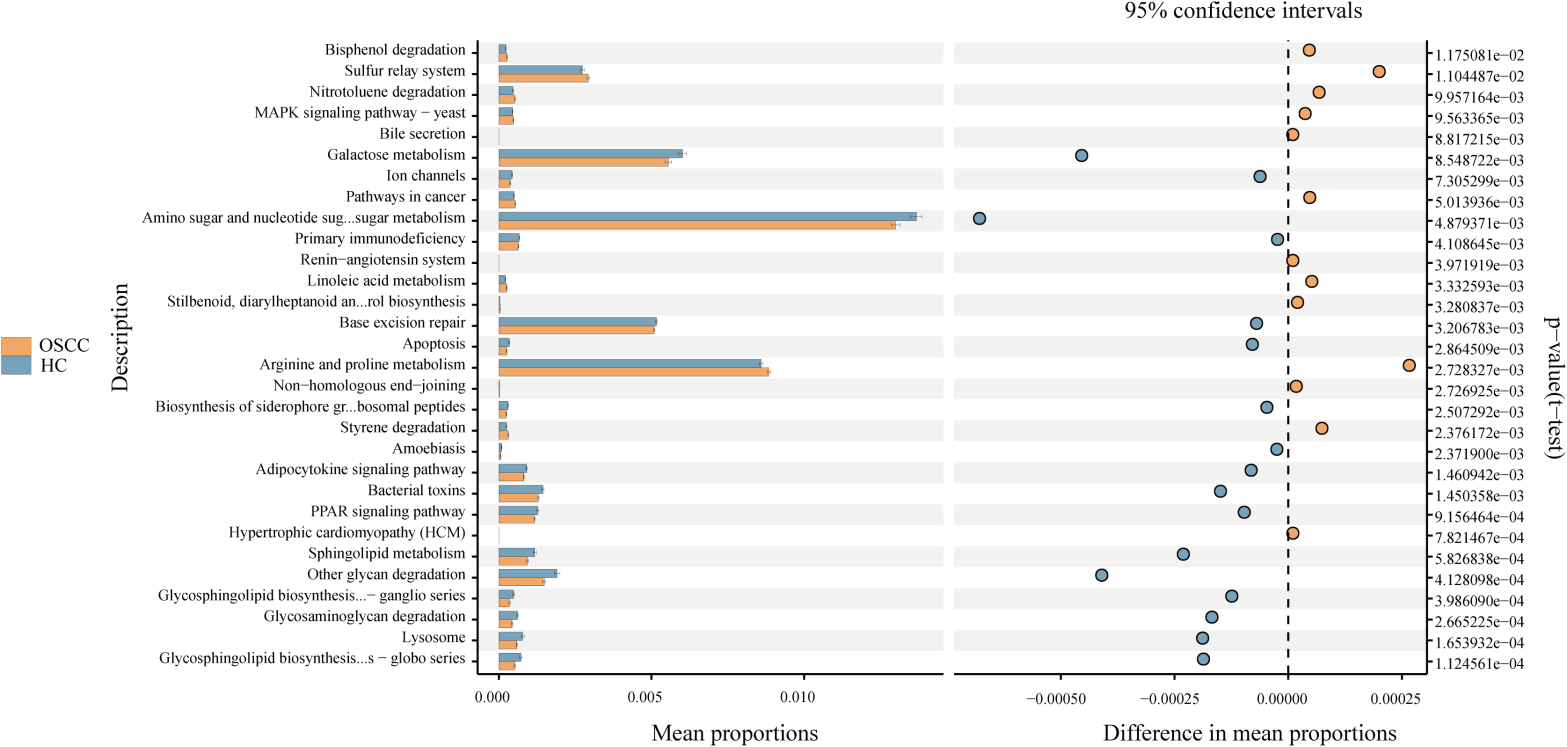
Differential functional pathways of bacteria in three groups predicted by PICRUSt2. All pathways represented here were analyzed using a double-sided Welch’s t-test with a significant P value of <0.05.

Using PICRUSt2 and the Kyoto Encyclopedia of Genes and Genomes database, the group observed differences in microbial functional pathways between the two groups. In the OSCC group, the microbes were predicted to be significantly more functional in arginine and proline metabolism, sulphur transfer system, styrene degradation, and linoleic acid metabolism (P<0.05). While in the control group the microbes were predicted to be significantly more functional in aminosugar and nucleotide sugar metabolism, galactose metabolism, other sugar degradation, and sphingolipid metabolism (P<0.05) (**Figure 4**).

### 3.6 Significant immune infiltration in tumor tissues of OSCC patients

**Figure 5:**
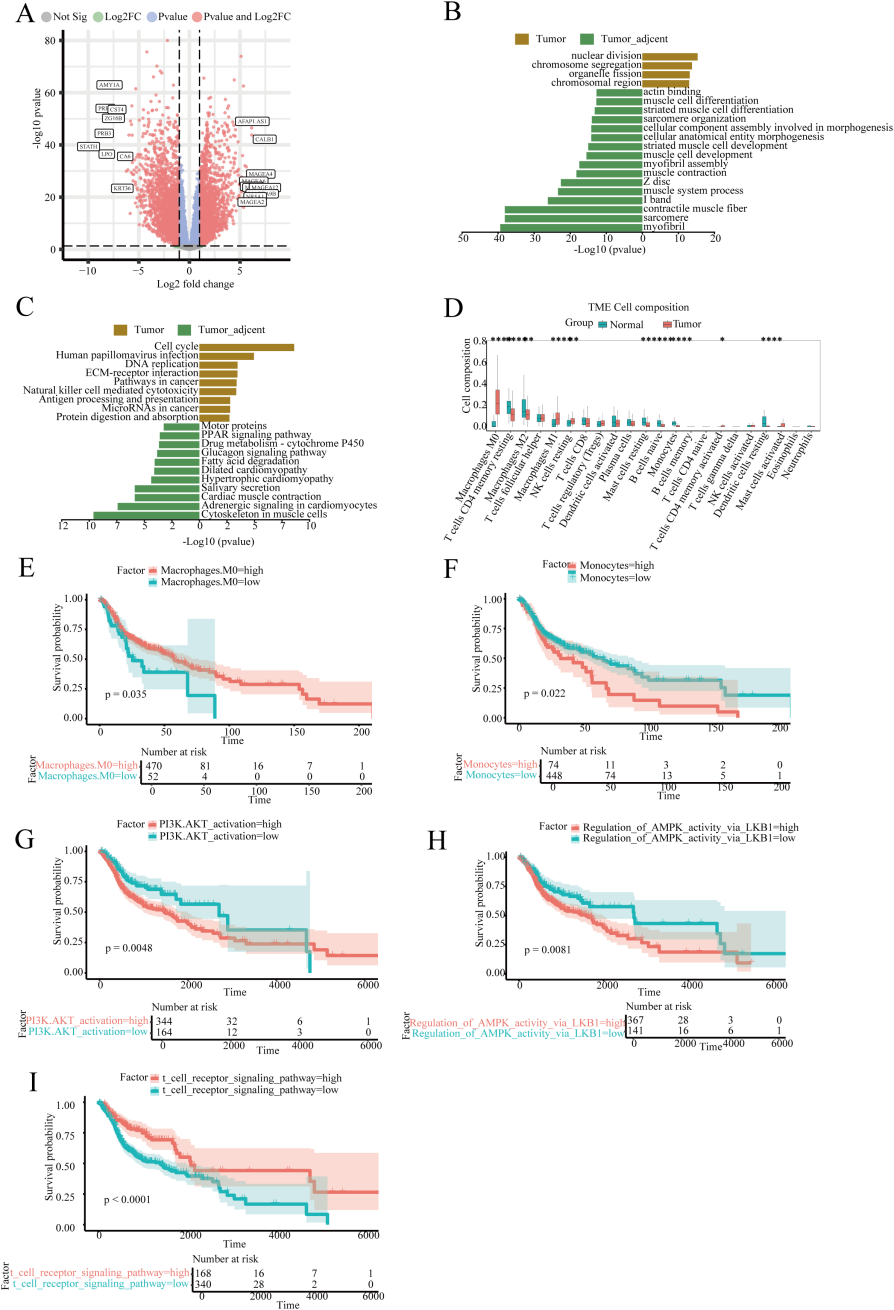
Transcriptome differential analysis, immune infiltration, and survival analysis of head and neck squamous cell carcinoma. **(A)** Volcano plot of differentially expressed genes; **(B, C)** GSEA enrichment analysis of differentially expressed genes in the GO and KEGG databases; **(D)** Boxplot analysis of the proportion of various immune cells in samples; **(E)** Survival analysis of the proportion of Macrophages cell subsets in tumor patients; **(F)** Survival analysis focusing on the proportion of Monocytes cell subsets among tumor patients; **(G)** Survival analysis based on the PI3K.AKT_activation signaling pathway score; **(H)** Survival analysis based on the Regulation_of_AMPK_activity_via_LKB1 signaling pathway score; **(I)** Survival analysis based on the t cell receptor signaling pathway signaling pathway score.

The DESeq2 software was used to analyses transcriptome data from TCGA database, with marked differentially expressed genes identified by applying a threshold of fold change > 2 and adjusted p-value (padj) < 0.05. A total of 4,105 significant differentially expressed genes were identified. **Figure 5A** illustrates the landscape of these genes using a volcano plot, highlighting the top 20 most upregulated and downregulated genes. GO and KEGG enrichment analysis revealed activation of cell cycle, DNA replication, and immune-related pathways such as Natural killer cell mediated cytotoxicity (**Figures 5B, C**).

Immune infiltration analysis indicated that macrophages, CD4 T cells, and T helper cells accounted for over 50% of the tumor tissue composition. The abundance of M0 and M1 macrophages in tumor tissues was significantly higher compared to normal tissues, whereas monocytes showed a marked decrease in tumor tissues (**Figure 5D**). Survival analysis suggested that a high abundance of M0 macrophages is associated with improved patient survival, whereas the survival impact of monocytes was inverse (**Figures 5E, F**).

Gene Set Variation Analysis (GSVA) results were utilized for survival analysis. Three common immune signaling pathways in tumors, PI3K.AKT_activation and Regulation_of_AMPK_activity_via_LKB1, showed that high enrichment was significantly associated with reduced patient survival. In contrast, high enrichment of the T cell receptor signaling pathway was associated with improved patient survival (**Figures 5G-I**).

### 3.7 The impact of tumor microbiota on tumor immune microenvironment in OSCC patients

**Figure 6:**
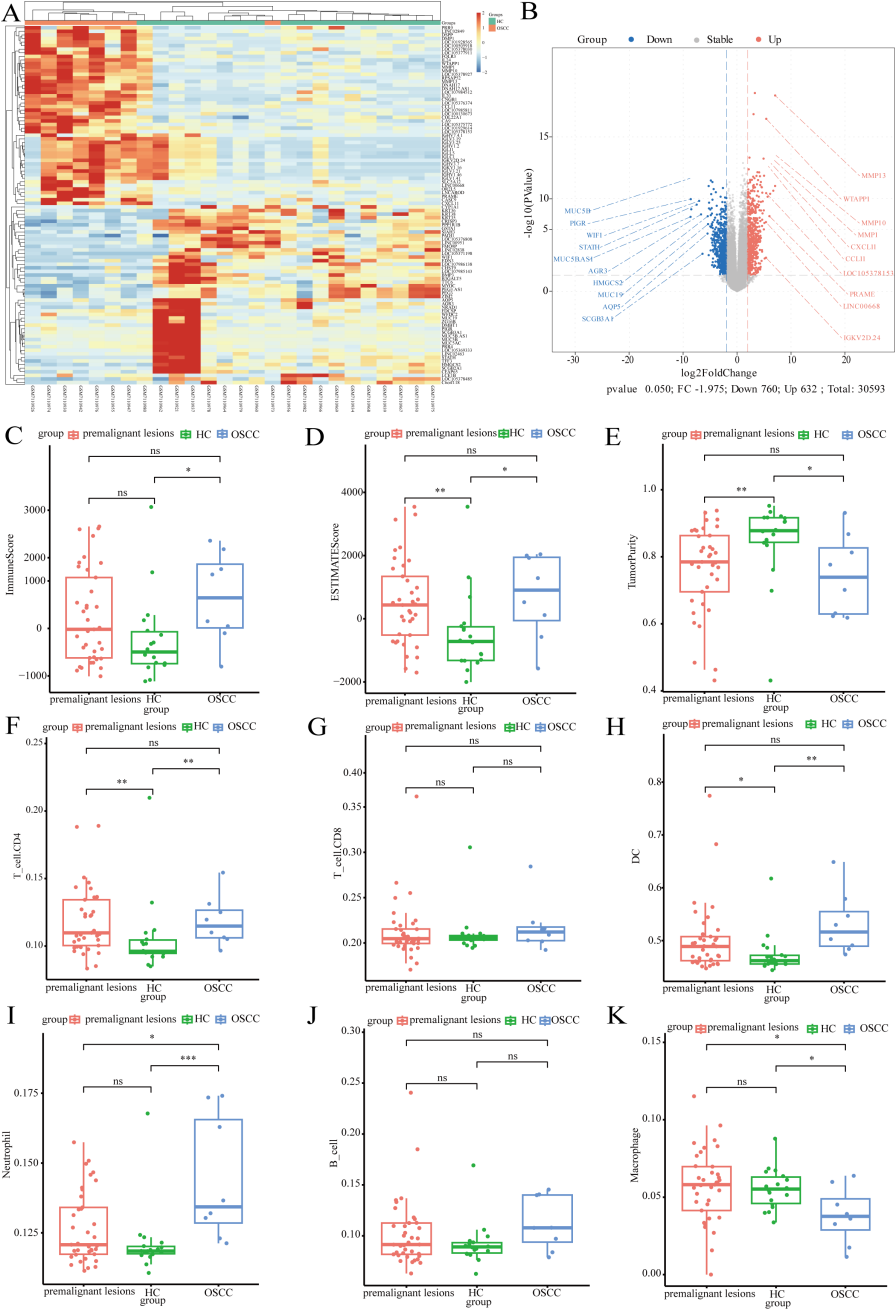
The impact of tumor microorganisms on the host transcriptome. **(A)** Heatmap of differentially expressed genes; **(B)** Volcano plot illustrating the differentially expressed genes; **(C)** Boxplot of immune scores across different groups; **(D)** Boxplot of ESTIMATE scores for varying groups **(E)** Boxplot depicting tumor purity in distinct groups; **(F)** Boxplot showing the relative abundance of T_cell.CD4 across groups; **(G)** Boxplot displaying the relative abundance of T_cell.CD8 among groups; **(H)** Boxplot of dendritic cells (DCs) relative abundance in different groups; **(I)** Boxplot illustrating neutrophil relative abundance by group; **(J)** Boxplot of B cell relative abundance in various groups; **(K)** Boxplot representing macrophage relative abundance across groups.

In this study, transcriptomics data from the GSE227919 dataset were downloaded and analyzed for differences (31). Heatmaps and volcano plots were used to illustrate the landscape of significantly differentially expressed genes between tumor and control groups (**Figures 6A, B**). Subsequently, we conducted immune scoring analysis, revealing that both the tumor and precancerous lesion groups exhibited higher ImmuneScores compared to the control group, with a similar trend observed in ESTIMATEScores (**Supplementary Figures**).

Tumor Purity was found to be lower in the tumor and precancerous lesion groups (**Figures 6C-E**). Immune infiltration analysis indicated that the abundance of CD4 T cells was significantly higher in both the tumor and precancerous lesion groups than in the control group, whereas no marked differences were observed in the abundance of CD8 T cells and B cells among the three groups (**Figures 6F, G, J**). Dendritic cells (DCs) accounted for approximately 50% of immune cells, with their abundance significantly higher in both the tumor and precancerous lesion groups compared to the control group. The abundance of neutrophils increased significantly with disease progression (**Figures 6H, I**). Macrophages constituted a lower proportion of immune cells, and their abundance was significantly higher in the control group, potentially associated with the polarization of macrophages under microbial stimulation (**Figure 6K**).

## 4 Discussion

In this study, microbiome analysis revealed preserved α-diversity across groups but significant β-diversity divergence, indicating distinct inter-group community structures despite comparable species richness. Demographic-geographic heterogeneities modulate oral microbiome dysbiosis patterns in OSCC, as evidenced by Dijk’s meta-analysis of 423 studies demonstrating predominant α-diversity elevation with significant β-diversity shifts in most cohorts, contrasted by paradoxical α-diversity depletion observed in geographically distinct populations (e.g., Indian subcontinent cohorts) (32). However, there were more than 45% ASVs in both groups, suggested that certain microbial taxa were shared between OSCC and healthy controls, which supports the hypothesis that OSCC does not cause a complete change in the oral microbial community, but may affect the abundance of specific taxa (20, 33–35). Recent studies report nuanced microbial alterations in OSCC, characterized by shifts in specific bacterial taxa rather than overall diversity (36, 37). This underscores the complexity of oral microbiome dysbiosis in carcinogenesis, necessitating focused investigation into taxonomic-specific variations and functional pathway modifications that may underlie OSCC pathogenesis.

Furthermore, our results indicated that the *Firmicutes* and *Bacteroidota* at the phylum level were similar between the two groups, which is in agreement with previous studies (38–40). However, the relative abundance of *Streptococcus* in the OSCC group at the genus level was significant lower than HC groups, including K. Hashimoto et al. have reported that the abundance of *Streptococcus* in the oral microbiota of OSCC was significantly reduced in both saliva and tissue samples compared to that of healthy individuals or patients with oral leukoplakia (41–44). However, Han conducted a large-scale meta-analysis by integrating 11 publicly available datasets and found an enrichment of *Streptococcus* spp. in OSCC (45). Methodological heterogeneity, including variations in saliva collection protocols (e.g., fasting vs. non-fasting) and cohort demographics (e.g., geographic differences), may account for these discrepancies. Future studies should adopt standardized protocols to minimize confounding. The results of LEfSe analyses showed that *Actinobacteriota* at the phylum level was significantly lower compared to HC groups. While Spirito and Ke Yang et al. similarly found a reduction in *Actinobacteriota* in OSCC saliva, which suggested it may plays an important role in the progression of OSCC (39, 46). Furthermore, this study also found *Capnocytophaga* was significant enrichment in OSCC group, previous studies have indicated that *Capnocytophaga*, especially *Capnocytophaga gingivalis*, was significantly enrichment in OSCC compared to healthy controls. It might be involved in pro-carcinogenic behaviors, such as invasion and metastasis, through mechanisms such as epithelial mesenchymal transition (46–48).

The significant correlation between oral microbiota and clinical features of OSCC suggests that the oral microbiota may be related to the progression and clinical features of OSCC. Phylum-level analysis demonstrated significant negative correlation between *Deferribacterota* abundance and neurotropic infiltration, suggesting this taxon’s potential protective role against perineural invasion in OSCC pathogenesis. Neuroinvasion is an important prognostic indicator of OSCC and is an independent risk factor for poor survival and cervical lymph node metastasis, especially intraneural invasion, in which tumor cells invade the neural structures, which is significantly associated with poorer cancer-specific survival (49, 50). In contrast, *Acidobacteriota* was positively correlated with the progression of T factor, which may imply that this phylum is associated with advanced stages of OSCC and may play a role in tumor growth and progression. At the genus level, *Collinsella* and *Catenibacterium* were positively correlated to betel nut chewing, which is a known risk factor for OSCC (51). Li (52) found that betel nut chewing could significantly affect the level of intestinal microorganisms, and *Collinsella* bacteria were found to be in higher abundance in colorectal cancer and esophageal cancer (53, 54).

*Candidatus Stoquefichus* has a certain positive correlation with clinical stage and N factor but it has been less studied in cancer. The study hypothesized that *Candidatus Stoquefichus* may be closely related to the progression of OSCC. *Lactococcus* was negative correlation with Neurological violation, but this result has only been studied at the correlation level. *Lactococcus lactis*, a member of this genus, was able to inhibit the proliferation of tumor cells through a variety of mechanisms, including induction of interleukin-18 expression, regulation of angiogenesis, direct induction of apoptosis, and alteration of metabolic preferences of macrophages (55–57). Limited by our experimental facilities, the mechanism of lactobacillus-host interactions has not been carried out. These findings underscore the multifaceted interplay between oral microbiome dysbiosis and OSCC pathogenesis. The results of KEGG enrichment found that arginine, proline metabolism, sulfur relay system, styrene degradation and linoleic acid metabolism, were significantly enriched in the OSCC group. These pathways play key roles in cellular metabolism and inflammation, and their dysregulation has been linked to cancer biology. The glutamine-arginine-proline metabolic axis is critical in cancer metabolism and serves as a scaffold for the synthesis of other amino acids and metabolites. This axis is involved in the regulation of amino acid metabolism, which is frequently altered in cancer cells to support rapid growth and proliferation (58). Whereas the Sulfur relay system plays an important role in cancer and is involved in the epigenetic regulation of cellular redox reactions, signaling, and gene expression, sulfur amino acid-related metabolism and vitamin B6-binding activity have been found to be down-regulated in hepatocellular carcinoma, suggesting that reprogramming of sulfur metabolism may be associated with tumor growth and survival (59, 60). Linoleic acid derivatives in Linoleic acid metabolism regulate inflammatory processes by affecting cell adhesion molecules and the transcription factor peroxisome proliferator-activated receptor pathway, which may influence cancer progression (61). However, Styrene degradation has been less studied in relation to cancer. These findings suggested that changes in OSCC microorganisms were not limited to taxonomic changes but also involved functional alterations that may affect tumor microenvironmental conditions and cancer progression. These results are consistent with recent findings that microbe-driven metabolic pathways can influence cancer development and progression (62).

The relationship between microorganisms and tumors is intricate and multifaceted. Research indicated that intra-tumor microorganisms may influence the production of cytokines, induce pro-inflammatory responses, and subsequently activate pathways such as NF-κB or STAT3, promoting tumor progression (63).Triner et al. reported that bacteria within tumors induce the production of IL-17, facilitating B-cell infiltration and tumor development (64). Additionally, microorganisms can induce oxidative/nitrosative DNA damage, leading to tumorigenesis, although data on this impact are limited (65). Activation of carcinogenic pathways is another role of intra-tumor microorganisms. Researchers have found that certain intra-tumor microorganisms can affect the production and secretion of cytokines, such as IL-6 and TNF-α (64). Intra-tumor microorganisms also impact the tumor’s immune microenvironment, thereby influencing tumor occurrence and cancer treatment.

This study observed a marked increase in the abundance of immune cells within tumor tissues of patients with OSCC, accompanied by a corresponding elevation in immune scores as the disease advanced. This phenomenon may be attributed to the activation of microorganisms resident within the carcinoma tissues, which appears to augment immune cell infiltration and precipitate alterations within the tumor’s immune microenvironment. Gene Set Enrichment Analysis (GSEA) of the upregulated genes in tumors uncovered significant activation of multiple signaling pathways that are pivotal to tumor immunity. These pathways include the INTERFERON_GAMMA_RESPONSE, ALLOGRAFT_REJECTION, INTERFERON_ALPHA_RESPONSE, INFLAMMATORY_RESPONSE, EPITHELIAL_MESENCHYMAL_TRANSITION, TNFA_SIGNALING_VIA_NFKB, E2F_TARGETS, G2M_CHECKPOINT, IL6_JAK_STAT3_SIGNALING, COMPLEMENT, and IL2_STAT5_SIGNALING (See **Supplementary Table 3** for details), indicating a complex interplay of immune responses in the tumor microenvironment.

The analysis of transcriptomic data from OSCC in the TCGA database indicated that signal pathways and terms related to cell cycle, mitosis, and chromosome segregation were significantly enriched in the tumor group, and different immune cell types have varying impacts on tumor survival. The abundance of M0 macrophages contributes to patient survival, which may be due to M0 macrophages being stimulated to transform into M1 macrophages, activating T cells through the t cell receptor signaling pathway to further enhance anti-tumor immune responses. Li et al. found important interactions between the oral microbiome, systemic diseases, immunity and cancer by counting clinical studies of the oral microbiome over the last decade (66).There is growing evidence that oral microbiome dysbiosis and specific microorganisms may play an important role in the onset, development, progression, and metastasis of OSCC through direct or indirect actions (67).

A limitation of this study was the relatively small sample size of both the OSCC group and the HC group, which may limit the generalizability of the findings. Although this study observed significant differences in microbiota composition and functional pathways between the two groups, larger cohort studies are needed to confirm these associations and to determine the robustness and reproducibility of the observed microbial profiles. Second, the cross-sectional design of this study did not allow for the identification of a causal relationship between alterations in the oral microbiota and the development of OSCC. Longitudinal studies are needed to track changes in the microbiome over time and to examine whether microbiota dysbiosis precedes or follows the onset of OSCC. Furthermore, while 16S rDNA sequencing provides valuable information about microbial composition, it does not provide a comprehensive picture of the functional capacity or metabolic activity of the microbiota. Although this study used PICRUSt2 to predict functional pathways, the accuracy of such predictions depends on existing reference databases and may not fully reflect the complexity of microbial function in the OSCC microenvironment. In addition, environmental factors such as diet, smoking, and alcohol consumption are known to influence the oral microbiome, but the present study did not comprehensively control for these factors and thus may confound the observed associations. Finally, our study focused primarily on bacterial taxa, whereas oral fungi, viruses, and other microbial communities may also play important roles in OSCC pathogenesis. Future studies combining metagenomic and multi genomic approaches will provide a more comprehensive understanding of the oral microbiota in OSCC.

## 5 Conclusion

This study elucidated the salivary microbiota in OSCC patients and microbiota-host interactions, establishing a theoretical framework for developing microbiota-based diagnostics and targeted therapies in OSCC, which could potentially enhance clinical guidance and serve as biomarkers or immunotherapeutic targets. Further studies on the functions and mechanisms of oral microbes through metabolite regulation, epigenetic regulation, single cell microbiomics and humanized mouse models are needed in the future to validate their role in OSCC pathogenesis and their potential as therapeutic targets.

## Data Availability

The datasets presented in this study can be found in online repositories. The names of the repository/repositories and accession number(s) can be found below: https://www.ncbi.nlm.nih.gov/, PRJNA1215301.
