# Supplementary figures and images for: Oral and intratumoral microbiota influence tumor immunity and patient survival
Source: Front Immunol. 2025 May 21;16:1572152. doi: 10.3389/fimmu.2025.1572152 (PMC12138198; doi:10.3389/fimmu.2025.1572152)

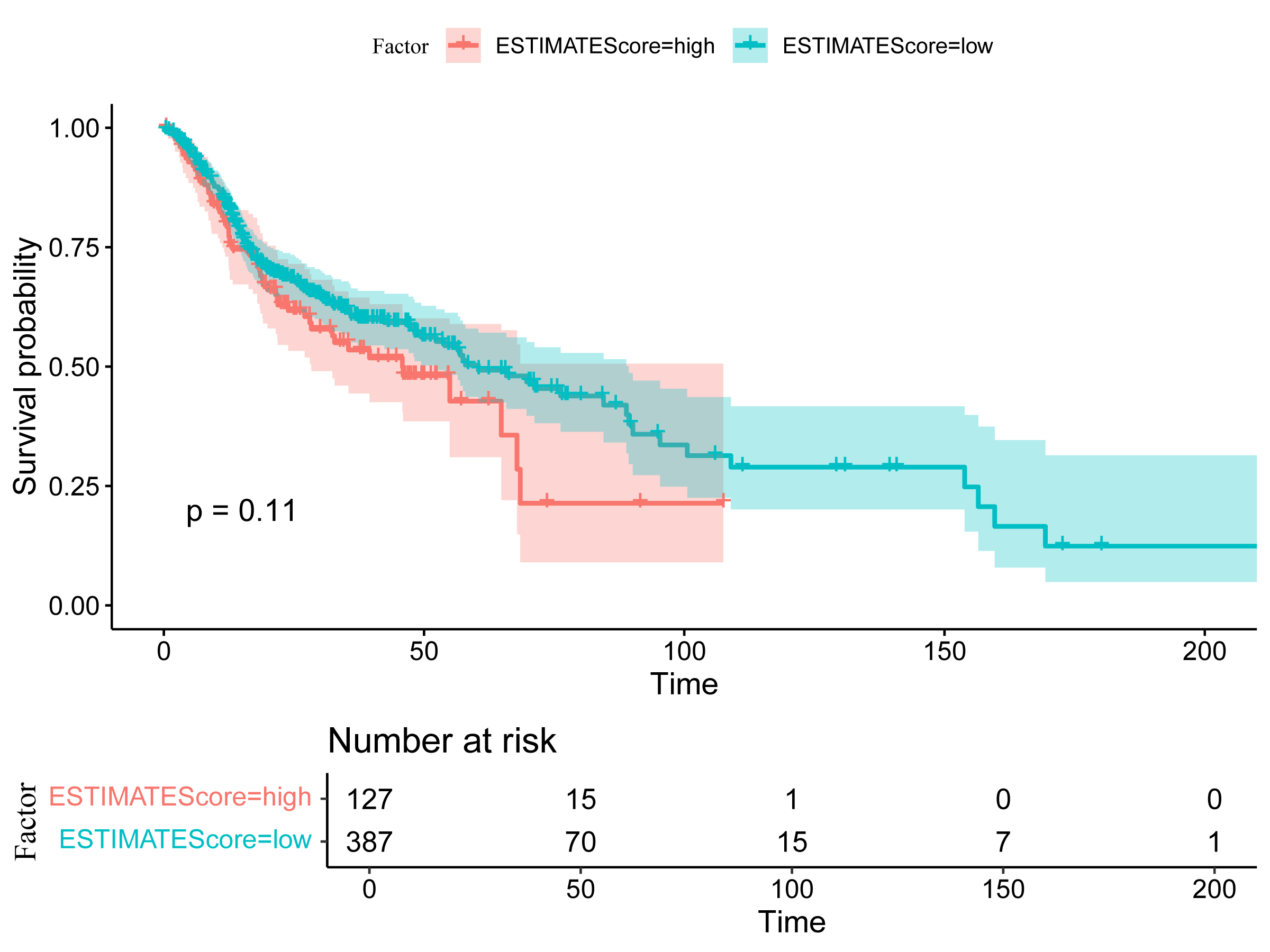

Supplement: Supplementary file 1 [file Image1.tif]

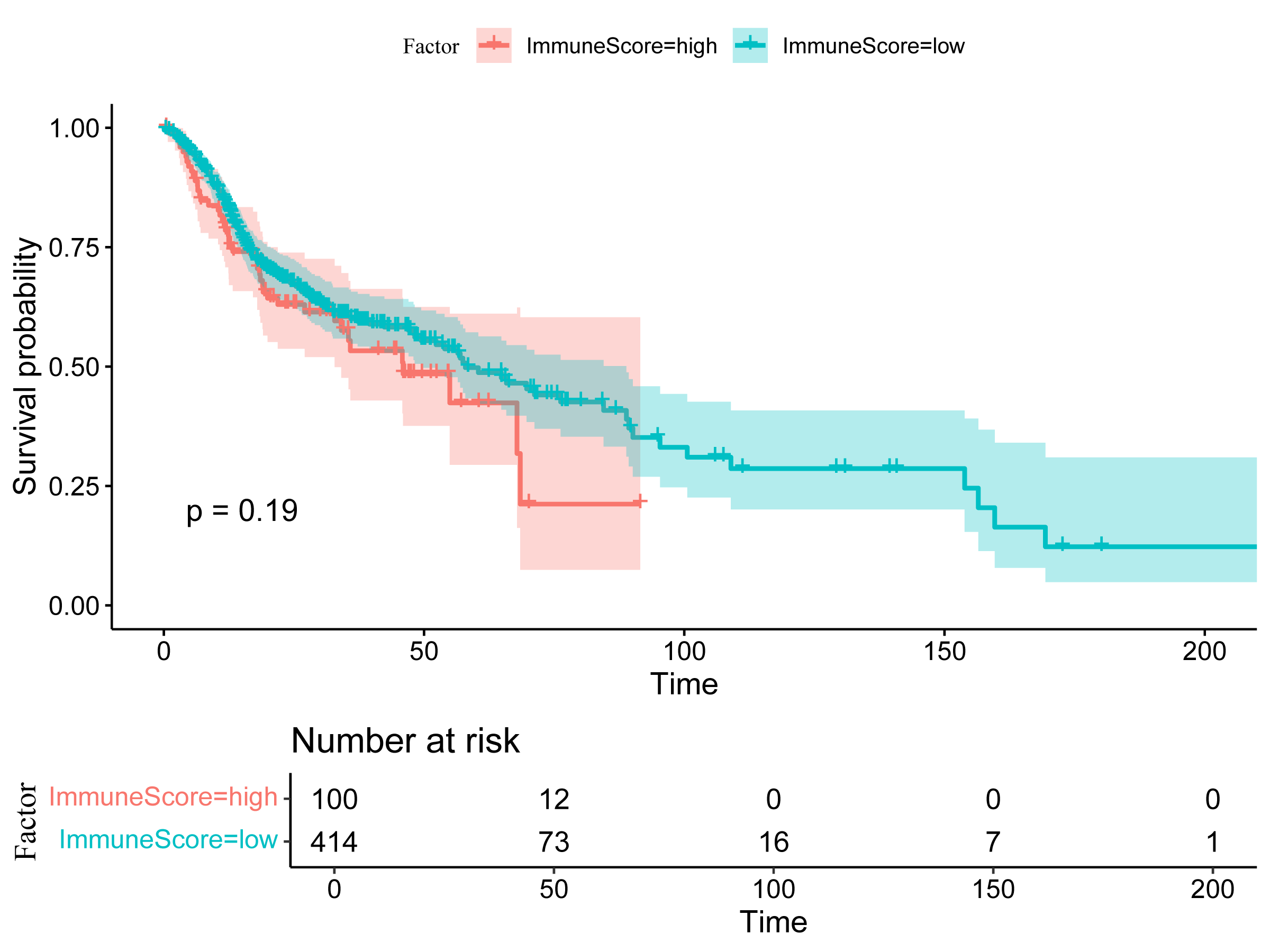

Supplement: Supplementary file 2 [file Image2.tif]

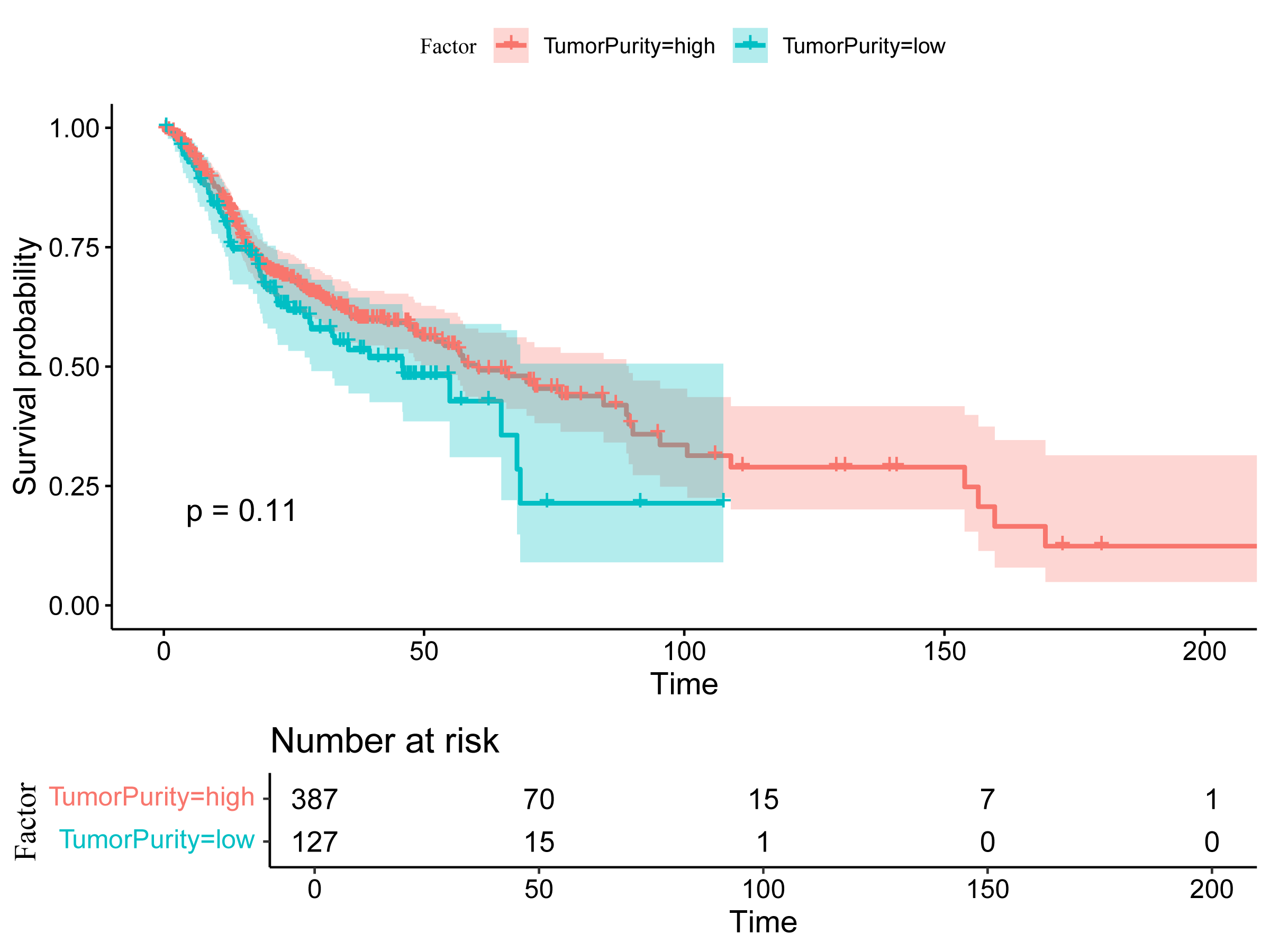

Supplement: Supplementary file 3 [file Image3.tif]
