# Supplementary material for: Oral and intratumoral microbiota influence tumor immunity and patient survival
Source: Front Immunol. 2025 May 21;16:1572152. doi: 10.3389/fimmu.2025.1572152 (PMC12138198; doi:10.3389/fimmu.2025.1572152)
Supplement: Supplementary file 4 [file Table1.docx]

Differentiation degree

| name | levels | 0 (N=3) | 1 (N=6) | 2 (N=24) | 3 (N=3) | p |
| --- | --- | --- | --- | --- | --- | --- |
| Age | Mean ± SD | 68.7 ± 6.5 | 69.8 ± 11.1 | 54.9 ± 15.4 | 45.0 ± 14.7 | .040 |
| BMI | Mean ± SD | 22.4 ± 3.9 | 24.4 ± 4.5 | 23.3 ± 3.8 | 27.4 ± 1.5 | .328 |
| Smoking | 0 | 3 (100%) | 6 (100%) | 12 (50%) | 2 (66.7%) | .069 |
|  | 1 | 0 (0%) | 0 (0%) | 12 (50%) | 1 (33.3%) |  |
| Drinking | 0 | 3 (100%) | 6 (100%) | 17 (70.8%) | 2 (66.7%) | .327 |
|  | 1 | 0 (0%) | 0 (0%) | 7 (29.2%) | 1 (33.3%) |  |
| Betel.nut.chewing | 0 | 3 (100%) | 6 (100%) | 20 (83.3%) | 3 (100%) | .522 |
|  | 1 | 0 (0%) | 0 (0%) | 4 (16.7%) | 0 (0%) |  |
| Stage | 1 | 0 (0%) | 1 (16.7%) | 5 (20.8%) | 2 (66.7%) | .104 |
|  | 2 | 2 (66.7%) | 5 (83.3%) | 7 (29.2%) | 0 (0%) |  |
|  | 3 | 1 (33.3%) | 0 (0%) | 3 (12.5%) | 0 (0%) |  |
|  | 4 | 0 (0%) | 0 (0%) | 9 (37.5%) | 1 (33.3%) |  |
| Lymph.node.metastasis | 0 | 3 (100%) | 6 (100%) | 14 (58.3%) | 2 (66.7%) | .147 |
|  | 1 | 0 (0%) | 0 (0%) | 10 (41.7%) | 1 (33.3%) |  |
| Nerve.invasion | 0 | 3 (100%) | 6 (100%) | 16 (66.7%) | 1 (33.3%) | .107 |
|  | 1 | 0 (0%) | 0 (0%) | 8 (33.3%) | 2 (66.7%) |  |
| Gender | female | 2 (66.7%) | 6 (100%) | 9 (37.5%) | 1 (33.3%) | .043 |
|  | male | 1 (33.3%) | 0 (0%) | 15 (62.5%) | 2 (66.7%) |  |

Drinking

| name | levels | 0 (N=28) | 1 (N=8) | p |
| --- | --- | --- | --- | --- |
| Age | Mean ± SD | 59.7 ± 15.0 | 50.9 ± 16.7 | .161 |
| BMI | Mean ± SD | 23.9 ± 3.5 | 23.2 ± 5.2 | .664 |
| Smoking | 0 | 22 (78.6%) | 1 (12.5%) | .003 |
|  | 1 | 6 (21.4%) | 7 (87.5%) |  |
| Betel.nut.chewing | 0 | 26 (92.9%) | 6 (75%) | .436 |
|  | 1 | 2 (7.1%) | 2 (25%) |  |
| Differentiation.degree | 0 | 3 (10.7%) | 0 (0%) | .327 |
|  | 1 | 6 (21.4%) | 0 (0%) |  |
|  | 2 | 17 (60.7%) | 7 (87.5%) |  |
|  | 3 | 2 (7.1%) | 1 (12.5%) |  |
| Stage | 1 | 6 (21.4%) | 2 (25%) | .293 |
|  | 2 | 13 (46.4%) | 1 (12.5%) |  |
|  | 3 | 3 (10.7%) | 1 (12.5%) |  |
|  | 4 | 6 (21.4%) | 4 (50%) |  |
| Lymph.node.metastasis | 0 | 22 (78.6%) | 3 (37.5%) | .074 |
|  | 1 | 6 (21.4%) | 5 (62.5%) |  |
| Nerve.invasion | 0 | 23 (82.1%) | 3 (37.5%) | .041 |
|  | 1 | 5 (17.9%) | 5 (62.5%) |  |
| Gender | female | 17 (60.7%) | 1 (12.5%) | .045 |
|  | male | 11 (39.3%) | 7 (87.5%) |  |

Gender

| name | levels | female (N=18) | male (N=18) | p |
| --- | --- | --- | --- | --- |
| Age | Mean ± SD | 62.4 ± 15.3 | 53.0 ± 14.7 | .068 |
| BMI | Mean ± SD | 24.3 ± 3.8 | 23.1 ± 4.0 | .364 |
| Smoking | 0 | 17 (94.4%) | 6 (33.3%) | <.001 |
|  | 1 | 1 (5.6%) | 12 (66.7%) |  |
| Drinking | 0 | 17 (94.4%) | 11 (61.1%) | .045 |
|  | 1 | 1 (5.6%) | 7 (38.9%) |  |
| Betel.nut.chewing | 0 | 18 (100%) | 14 (77.8%) | .112 |
|  | 1 | 0 (0%) | 4 (22.2%) |  |
| Differentiation.degree | 0 | 2 (11.1%) | 1 (5.6%) | .043 |
|  | 1 | 6 (33.3%) | 0 (0%) |  |
|  | 2 | 9 (50%) | 15 (83.3%) |  |
|  | 3 | 1 (5.6%) | 2 (11.1%) |  |
| Stage | 1 | 3 (16.7%) | 5 (27.8%) | .564 |
|  | 2 | 9 (50%) | 5 (27.8%) |  |
|  | 3 | 2 (11.1%) | 2 (11.1%) |  |
|  | 4 | 4 (22.2%) | 6 (33.3%) |  |
| Lymph.node.metastasis | 0 | 15 (83.3%) | 10 (55.6%) | .148 |
|  | 1 | 3 (16.7%) | 8 (44.4%) |  |
| Nerve.invasion | 0 | 14 (77.8%) | 12 (66.7%) | .710 |
|  | 1 | 4 (22.2%) | 6 (33.3%) |  |

Lymph node metastasis

| name | levels | 0 (N=25) | 1 (N=11) | p |
| --- | --- | --- | --- | --- |
| Age | Mean ± SD | 59.6 ± 14.5 | 53.4 ± 17.7 | .271 |
| BMI | Mean ± SD | 24.5 ± 3.7 | 21.9 ± 3.9 | .058 |
| Smoking | 0 | 19 (76%) | 4 (36.4%) | .057 |
|  | 1 | 6 (24%) | 7 (63.6%) |  |
| Drinking | 0 | 22 (88%) | 6 (54.5%) | .074 |
|  | 1 | 3 (12%) | 5 (45.5%) |  |
| Betel.nut.chewing | 0 | 22 (88%) | 10 (90.9%) | 1.000 |
|  | 1 | 3 (12%) | 1 (9.1%) |  |
| Differentiation.degree | 0 | 3 (12%) | 0 (0%) | .147 |
|  | 1 | 6 (24%) | 0 (0%) |  |
|  | 2 | 14 (56%) | 10 (90.9%) |  |
|  | 3 | 2 (8%) | 1 (9.1%) |  |
| Stage | 1 | 8 (32%) | 0 (0%) | <.001 |
|  | 2 | 14 (56%) | 0 (0%) |  |
|  | 3 | 1 (4%) | 3 (27.3%) |  |
|  | 4 | 2 (8%) | 8 (72.7%) |  |
| Nerve.invasion | 0 | 20 (80%) | 6 (54.5%) | .243 |
|  | 1 | 5 (20%) | 5 (45.5%) |  |
| Gender | female | 15 (60%) | 3 (27.3%) | .148 |
|  | male | 10 (40%) | 8 (72.7%) |  |

Nerve invasion

| name | levels | 0 (N=26) | 1 (N=10) | p |
| --- | --- | --- | --- | --- |
| Age | Mean ± SD | 60.0 ± 16.8 | 51.9 ± 10.4 | .167 |
| BMI | Mean ± SD | 23.9 ± 3.7 | 23.4 ± 4.5 | .732 |
| Smoking | 0 | 19 (73.1%) | 4 (40%) | .143 |
|  | 1 | 7 (26.9%) | 6 (60%) |  |
| Drinking | 0 | 23 (88.5%) | 5 (50%) | .041 |
|  | 1 | 3 (11.5%) | 5 (50%) |  |
| Betel.nut.chewing | 0 | 24 (92.3%) | 8 (80%) | .645 |
|  | 1 | 2 (7.7%) | 2 (20%) |  |
| Differentiation.degree | 0 | 3 (11.5%) | 0 (0%) | .107 |
|  | 1 | 6 (23.1%) | 0 (0%) |  |
|  | 2 | 16 (61.5%) | 8 (80%) |  |
|  | 3 | 1 (3.8%) | 2 (20%) |  |
| Stage | 1 | 5 (19.2%) | 3 (30%) | .120 |
|  | 2 | 13 (50%) | 1 (10%) |  |
|  | 3 | 3 (11.5%) | 1 (10%) |  |
|  | 4 | 5 (19.2%) | 5 (50%) |  |
| Lymph.node.metastasis | 0 | 20 (76.9%) | 5 (50%) | .243 |
|  | 1 | 6 (23.1%) | 5 (50%) |  |
| Gender | female | 14 (53.8%) | 4 (40%) | .710 |
|  | male | 12 (46.2%) | 6 (60%) |  |

Smoking

| name | levels | 0 (N=23) | 1 (N=13) | p |
| --- | --- | --- | --- | --- |
| Age | Mean ± SD | 64.1 ± 14.5 | 46.4 ± 10.2 | <.001 |
| BMI | Mean ± SD | 24.1 ± 3.8 | 23.0 ± 4.1 | .433 |
| Drinking | 0 | 22 (95.7%) | 6 (46.2%) | .003 |
|  | 1 | 1 (4.3%) | 7 (53.8%) |  |
| Betel.nut.chewing | 0 | 23 (100%) | 9 (69.2%) | .023 |
|  | 1 | 0 (0%) | 4 (30.8%) |  |
| Differentiation.degree | 0 | 3 (13%) | 0 (0%) | .069 |
|  | 1 | 6 (26.1%) | 0 (0%) |  |
|  | 2 | 12 (52.2%) | 12 (92.3%) |  |
|  | 3 | 2 (8.7%) | 1 (7.7%) |  |
| Stage | 1 | 5 (21.7%) | 3 (23.1%) | .261 |
|  | 2 | 11 (47.8%) | 3 (23.1%) |  |
|  | 3 | 3 (13%) | 1 (7.7%) |  |
|  | 4 | 4 (17.4%) | 6 (46.2%) |  |
| Lymph.node.metastasis | 0 | 19 (82.6%) | 6 (46.2%) | .057 |
|  | 1 | 4 (17.4%) | 7 (53.8%) |  |
| Nerve.invasion | 0 | 19 (82.6%) | 7 (53.8%) | .143 |
|  | 1 | 4 (17.4%) | 6 (46.2%) |  |
| Gender | female | 17 (73.9%) | 1 (7.7%) | <.001 |
|  | male | 6 (26.1%) | 12 (92.3%) |  |

Stage

| name | levels | 1 (N=8) | 2 (N=14) | 3 (N=4) | 4 (N=10) | p |
| --- | --- | --- | --- | --- | --- | --- |
| Age | Mean ± SD | 60.8 ± 15.5 | 60.0 ± 13.4 | 65.2 ± 21.8 | 49.1 ± 14.5 | .205 |
| BMI | Mean ± SD | 26.3 ± 3.5 | 23.9 ± 3.8 | 24.0 ± 2.9 | 21.4 ± 3.6 | .055 |
| Smoking | 0 | 5 (62.5%) | 11 (78.6%) | 3 (75%) | 4 (40%) | .261 |
|  | 1 | 3 (37.5%) | 3 (21.4%) | 1 (25%) | 6 (60%) |  |
| Drinking | 0 | 6 (75%) | 13 (92.9%) | 3 (75%) | 6 (60%) | .293 |
|  | 1 | 2 (25%) | 1 (7.1%) | 1 (25%) | 4 (40%) |  |
| Betel.nut.chewing | 0 | 7 (87.5%) | 12 (85.7%) | 4 (100%) | 9 (90%) | .880 |
|  | 1 | 1 (12.5%) | 2 (14.3%) | 0 (0%) | 1 (10%) |  |
| Differentiation.degree | 0 | 0 (0%) | 2 (14.3%) | 1 (25%) | 0 (0%) | .104 |
|  | 1 | 1 (12.5%) | 5 (35.7%) | 0 (0%) | 0 (0%) |  |
|  | 2 | 5 (62.5%) | 7 (50%) | 3 (75%) | 9 (90%) |  |
|  | 3 | 2 (25%) | 0 (0%) | 0 (0%) | 1 (10%) |  |
| Lymph.node.metastasis | 0 | 8 (100%) | 14 (100%) | 1 (25%) | 2 (20%) | <.001 |
|  | 1 | 0 (0%) | 0 (0%) | 3 (75%) | 8 (80%) |  |
| Nerve.invasion | 0 | 5 (62.5%) | 13 (92.9%) | 3 (75%) | 5 (50%) | .120 |
|  | 1 | 3 (37.5%) | 1 (7.1%) | 1 (25%) | 5 (50%) |  |
| Gender | female | 3 (37.5%) | 9 (64.3%) | 2 (50%) | 4 (40%) | .564 |
|  | male | 5 (62.5%) | 5 (35.7%) | 2 (50%) | 6 (60%) |  |
