# Supplementary material for: Oral and intratumoral microbiota influence tumor immunity and patient survival
Source: Front Immunol. 2025 May 21;16:1572152. doi: 10.3389/fimmu.2025.1572152 (PMC12138198; doi:10.3389/fimmu.2025.1572152)
Supplement: Supplementary file 5 [file Table2.docx]

Supplementary Table 2 Enrichment results of differential species screened by LEfSe analysis in the two groups（LDA＞3.5）

| level | OSCC Group | HC Group |
| --- | --- | --- |
| Phylum | - | Actinobacteriota |
| Class | - | Actinobacteria |
| Order | Flavobacteriales | Bacteroidales、Actinomycetales、Micrococcales |
| Family | Flavobacteriaceae | Prevotellaceae、Actinomycetaceae、Micrococcaceae |
| Genus | Capnocytophaga | Streptococcus、Prevotella_7、Prevotella、Actinomyces、Rothia |
| Species | Capnocytophaga_unclassified | Prevotella_7_unclassified、Streptococcus_pneumoniae、Prevotella_nanceiensis、Actinomyces_unclassified、Rothia -unclassified |
